# Association of specific gene mutations derived from machine learning with survival in lung adenocarcinoma

**DOI:** 10.1371/journal.pone.0207204

**Published:** 2018-11-12

**Authors:** Han-Jun Cho, Soonchul Lee, Young Geon Ji, Dong Hyeon Lee

**Affiliations:** 1 Department of Physiology, CHA University School of Medicine, Gyeonggi, Republic of Korea; 2 Catholic University School of Medicine, Secho-gu, Seoul, Republic of Korea; 3 Department of Orthopaedic Surgery, CHA Bundang Medical Center, CHA University School of Medicine, Gyeonggi, Republic of Korea; 4 Department of Preventive Medicine, CHA University School of Medicine, Gyeonggi, Republic of Korea; National Cancer Center, JAPAN

## Abstract

Lung cancer is the second most common cancer in the United States and the leading cause of mortality in cancer patients. Biomarkers predicting survival of patients with lung cancer have a profound effect on patient prognosis and treatment. However, predictive biomarkers for survival and their relevance for lung cancer are not been well known yet. The objective of this study was to perform machine learning with data from The Cancer Genome Atlas of patients with lung adenocarcinoma (LUAD) to find survival-specific gene mutations that could be used as survival-predicting biomarkers. To identify survival-specific mutations according to various clinical factors, four feature selection methods (information gain, chi-squared test, minimum redundancy maximum relevance, and correlation) were used. Extracted survival-specific mutations of LUAD were applied individually or as a group for Kaplan-Meier survival analysis. Mutations in *MMRN2* and *GMPPA* were significantly associated with patient mortality while those in *ZNF560* and *SETX* were associated with patient survival. Mutations in *DNAJC2* and *MMRN2* showed significant negative association with overall survival while mutations in *ZNF560* showed significant positive association with overall survival. Mutations in *MMRN2* showed significant negative association with disease-free survival while mutations in *DRD3* and *ZNF560* showed positive associated with disease-free survival. Mutations in *DRD3*, *SETX*, and *ZNF560* showed significant positive association with survival in patients with LUAD while the opposite was true for mutations in *DNAJC2*, *GMPPA*, and *MMRN2*. These gene mutations were also found in other cohorts of LUAD, lung squamous cell carcinoma, and small cell lung cancer. In LUAD of Pan-Lung Cancer cohort, mutations in *GMPPA*, *DNAJC2*, and *MMRN2* showed significant negative associations with survival of patients while mutations in *DRD3* and *SETX* showed significant positive association with survival. In this study, machine learning was conducted to obtain information necessary to discover specific gene mutations associated with the survival of patients with LUAD. Mutations in the above six genes could predict survival rate and disease-free survival rate in patients with LUAD. Thus, they are important biomarker candidates for prognosis.

## Introduction

Lung cancer is the leading cause of death in patients with cancer. It is the second most common cancer in men and women to date in the United States, following prostate cancer in men and breast cancer in women, respectively [1–3]. In the 1990s, stomach and lung cancers were leading causes of death among cancer patients in Korea, with stomach cancer accounting for 25% of cases while lung cancer accounting for 17% of cases. In 2016, 17,963 people died from lung cancer in Korea, accounting for 23% of all cancer-related deaths. Although lung cancer has the highest mortality rate, few biomarkers for predicting overall survival or disease-free survival have been reported. Accurately predicting survival rate in patients with cancer has a significant impact on their prognosis and treatment [4–6].

Machine learning methods have been used in a variety of ways in cancer research [7–10]. These methods can be used to identify breast cancer patients by genetic mutations as a result of applying gene learning methods to breast cancer samples [7]. One prostate cancer study has combined machine learning methods with National Institute for Health and Care Excellence features to observe the association between the prognosis of prostate cancer and genetic mutation profile [8]. In addition, previous studies have applied machine learning using healthy eating index scores to predict the interaction between colorectal cancer and overweight status [9]. In another study, modeling was used to demonstrate the benefit of exact binomial test for analyzing genome-wide somatic gene mutation through performance comparisons among different machine learning models [10].

In this study, machine learning methods with data from The Cancer Genome Atlas (TCGA) of patients with lung adenocarcinoma (LUAD) were utilized to discover gene mutations associated with patient survival. Results suggested that mutations in six genes, *DRD3*, *SETX*, *ZNF560*, *DNAJC2*, *GMPPA*, and *MMRN2*, were significantly associated with survival and overall survival time of patients with LUAD. These gene mutations could be used as survival-predicting biomarkers. Machine learning can be a useful tool to discover important biomarkers for predicting prognosis and survival in patients with lung cancer.

## Materials and methods

### Ethics statement

All patient data were acquired from previously published studies where written informed consents were obtained [1,11–15]. The TCGA-LUAD cohort, Pan-Lung Cancer cohort, Lung Squamous Cell Carcinoma TCGA cohort, Small Cell Lung Cancer cohort, Lung Adenocarcinoma Broad cohort, and Lung Adenocarcinoma MSKCC cohort in their methods stated that “Specimens were obtained from patients, with appropriate consent from institutional review boards” [1], “All specimens were obtained from patients with appropriate consent and with approval from the relevant institutional review boards” [11], “All specimens were obtained from patients with appropriate consent from the relevant Institutional Review Board” [12], “Human tumour samples were obtained from patients under IRB-approved protocols following written informed consent” [13], “Informed consent (Institutional Review Board) was obtained for each sample using protocols approved by the Broad Institute of Harvard and MIT and each originating tissue source site” [14], and “All patients had consented to Institutional Review Board-approved protocols permitting tissue collection and sequencing” [15], respectively.

### Data resource

TCGA-LUAD provided data for LUAD patients with somatic non-silent mutations and clinical information. These TCGA data were downloaded and then divided into clinical data matrix and mutation data matrix. In the original data, clinical information of 471 patients and mutation status of about 40,000 genes were recorded. After preprocessing, label setting and identification were given. Among 471 patients in the data set, 303 living (64.3%) and 168 dead (35.7%) patients were divided accordingly into the survival and non-survival groups, respectively. Data sets of Pan-Lung Cancer cohort (n = 954), Lung Squamous Cell Carcinoma TCGA cohort (n = 498), Small Cell Lung Cancer cohort (n = 101), Lung Adenocarcinoma Broad cohort (n = 135), and Lung Adenocarcinoma MSKCC cohort (n = 34) were also used to determine specific gene mutation frequencies. The first three data sets were used for association of specific gene mutation with survival. All data used within this study were obtained from open access data sets. They have passed the criteria for unrestricted publication with the following statement listed at https://cancergenome.nih.gov/publications/publicationguidelines “No restrictions; all data available without limitations”.

### Machine learning

RapidMinor (Boston, MA, USA) was the software used for machine learning. For feature selection, information gain, Chi-squared test, minimum redundancy maximum relevance, and correlation algorithm were used. Classification algorithms included Naive Bayes, k-nearest neighbors, support vector machine, and decision trees [16]. This study concentrated on the yield and selection of gene mutations using dependent algorithms rather than improvement of algorithms (S1 Fig). Accuracy, precision, recall, classification error, and correlation are shown in S1 Table.

### Data analysis

For the specificity of gene mutations, Fisher’s exact test and Kaplan-Meier analysis were applied. Frequencies of gene mutations were compared using Fisher’s exact test. Overall survival and disease-free survival were calculated using Kaplan-Meier analysis based on clinical information of mortality, survival, and observation time for patients. cBioPortal software was used to evaluate gene mutation status within the TCGA-LUAD cohort [17, 18]. Statistical significant was considered when p value was less than 0.05.

## Results

**Fig 1:**
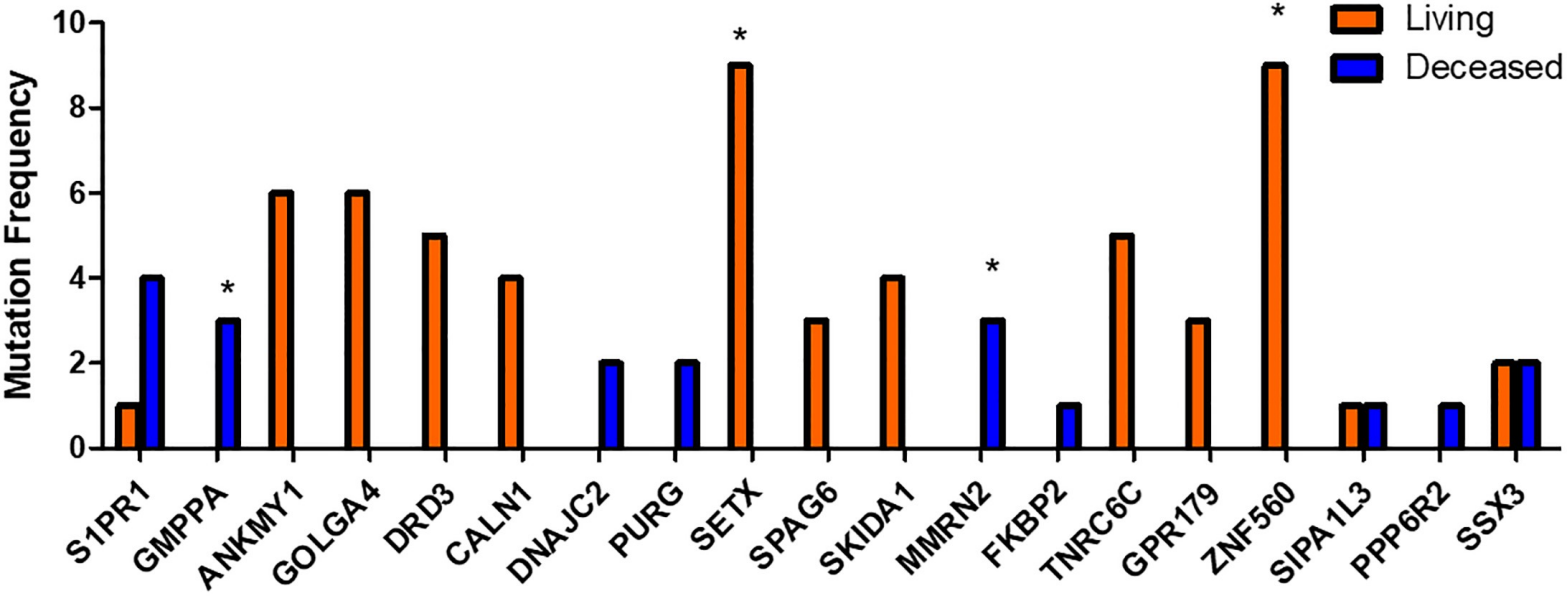
Comparison of mutation frequencies in 19 genes. Mutations in 19 genes were demonstrated as frequent and analyzed using Fisher’s extract test. * indicates p < 0.05.

In this study, to discover gene mutations predicting survival, data of LUAD patients obtained from TCGA were processed and classified using machine learning methods. Mutations in 19 genes were then selected and analyzed by frequencies, overall survival, and disease-free survival. Results suggested that specific gene mutations were associated with patient survival. Among mutations in 19 genes, mutations in *GMPPA* and *MMRN2* were significantly associated with patient mortality while mutations in *ZNF560* and *SETX* were significantly associated with patient survival (Fig 1 and Table 1). Mutations in *DNAJC2* and *MMRN2* showed significant negative association with overall survival while mutations in *ZNF560* showed significant positive association with overall survival. The median survival time in patients with LUAD was about 49 months. However, the median survival time in patients with mutations in *MMRN2* was about 11 months. Mutations in *MMRN2* were significantly and negatively associated with disease-free survival while mutations in *DRD3* and *ZNF560* were significantly and positively associated with disease-free survival. The median disease-free survival time in patients with LUAD was about 36 months while that in patients with mutations in *MMRN2* was about 5 months. Mutations in *DNAJC2*, *GMPPA*, *MMRN2*, *DRD3*, *SETX*, and *ZNF560* were associated with survival in patients with LUAD.

**Table 1:** Comparative analysis of mutation frequency and survival with mutations in 19 genes selected by feature selection methods.

Chromosome		1	2		3		7		8	9	10			11	17		19		22	X
Mutated gene		S1PR1	GMPPA	ANKMY1	GOLGA4	DRD3	CALN1	DNAJC2	PURG	SETX	SPAG6	SKIDA1	MMRN2	FKBP2	TNRC6C	GPR179	ZNF560	SIPA1L3	PPP6R2	SSX3
Mutation	Total	5	3	6	6	5	4	2	2	9	3	4	3	1	5	3	9	2	1	4
		(1.06%)	(0.64%)	(1.27%)	(1.27%)	(1.06%)	(0.85%)	(0.42%)	(0.42%)	(1.91%)	(0.64%)	(0.85%)	(0.64%)	(0.21%)	(1.06%)	(0.64%)	(1.91%)	(0.42%)	(0.21%)	(0.85%)
	Living	1	0	6	6	5	4	0	0	9	3	4	0	0	5	3	9	1	0	2
	Deceased	4	3	0	0	0	0	2	2	0	0	0	3	1	0	0	0	1	1	2
	Fisher’s exact	0.057	0.045*	0.930	0.930	0.166	0.302	0.127	0.127	0.030*	0.556	0.302	0.045*	0.357	0.166	0.556	0.030*	1.000	0.357	0.619
Overall Survival	P-value	0.154	0.061	0.209	0.132	0.075	0.380	6.66E-09***	0.158	0.113	0.307	0.123	4.20E-05***	0.311	0.086	0.467	0.032*	0.784	0.743	0.622
	Median Months	32.82	28.09	NA	NA	NA	NA	0	NA	NA	NA	NA	11.27	29.73	NA	NA	NA	46.68	49.01	9.95
Disease Free Survival	P-value	0.269	0.055	0.468	0.989	0.049*	0.245	NA	0.846	0.062	0.280	0.843	4.29E-05***	NA	0.479	0.333	0.034*	0.514	0.751	0.498
	Median Months	13.86	6.87	NA	22.60	NA	NA	NA	NA	NA	NA	84.57	4.57	NA	NA	NA	NA	17.90	35.58	18.66

Median month means the median survival time of patients in a given group. Fisher’s exact P-value: Log rank test, NA: Not Available,

* indicates p<0.05,

*** indicates p<0.001

As shown in Table 2, patients with LUAD lacking mutations in *DNAJC2* or *MMRN2* had median survival time of about 48 months while those with mutations in *DNAJC2* or *MMRN2* all died within 20 months (S2 Fig). About 42% of patients with LUAD lacking a mutation in *MMRN2* relapsed with a median disease-free time of about 33 months while those with mutations in *MMRN2* all relapsed within 10 months. Therefore, mutations in *DNAJC2* and/or *MMRN2* are considered to be predictors of survival or relapse of patients with LUAD since the probability of death or recurrence due to LUAD might be higher in the presence of mutations in *DNAJC2* or *MMRN2*. In contrast, patients with LUAD without a mutation in *ZNF560* had shorter survival than other patients, with a median survival time of about 45 months while those with mutations in *ZNF560* all survived. Additionally, about 42% of patients with LUAD without a mutation in *ZNF560* or *DRD3* relapsed with a median disease-free time of about 30 months. However, those with mutations in *ZNF560* or *DRD3* relapsed at a rate of about 11% or 0%, respectively. Because the probability of death or relapse due to LUAD might be lower when the *ZNF560* or *DRD3* was mutated, mutations in ZNF560 or DRD3 were considered to be predictors of survival or relapse in patients with LUAD.

**Table 2:** Comparative analysis of overall survival and disease-free survival in the gene mutation group and non-mutation group.

Survival Gene	Overall Survival									Disease Free Survival								
	P-value	Mutation Negative				Mutation Positive				P-value	Mutation Negative				Mutation Positive			
		Total Case	Decease Case (N)	Decease Case (%)	Median Survival (month)	Total Case	Decease Case (N)	Decease Case (%)	Median Survival (month)		Total Case	Relapse Case (N)	Relapse Case (%)	Median Disease Free (month)	Total Case	Relapse Case (N)	Relapse Case (%)	Median Disease Free (month)
GMPPA	0.061	468	165	35.26%	49.01	3	3	100.00%	28.09	0.055	399	166	41.60%	33.15	2	2	100.00%	6.87
DRD3	0.075	466	168	36.05%	46.68	5	0	0.00%	NA	0.049	396	168	42.42%	30.98	5	0	0.00%	NA
DNAJC2	0.0000000067	469	166	35.39%	47.77	2	2	100.00%	0	NA	NA	NA	NA	NA	NA	NA	NA	NA
SETX	0.113	462	168	36.36%	46.68	9	0	0.00%	NA	0.062	392	168	42.86%	30.98	9	0	0.00%	NA
MMRN2	0.000042	468	165	35.26%	47.77	3	3	100.00%	11.27	0.000043	399	166	41.60%	33.15	2	2	100.00%	4.57
ZNF560	0.032	462	168	36.36%	45.3	9	0	0.00%	NA	0.034	392	167	42.60%	30.39	9	1	11.11%	NA

**Fig 2:**
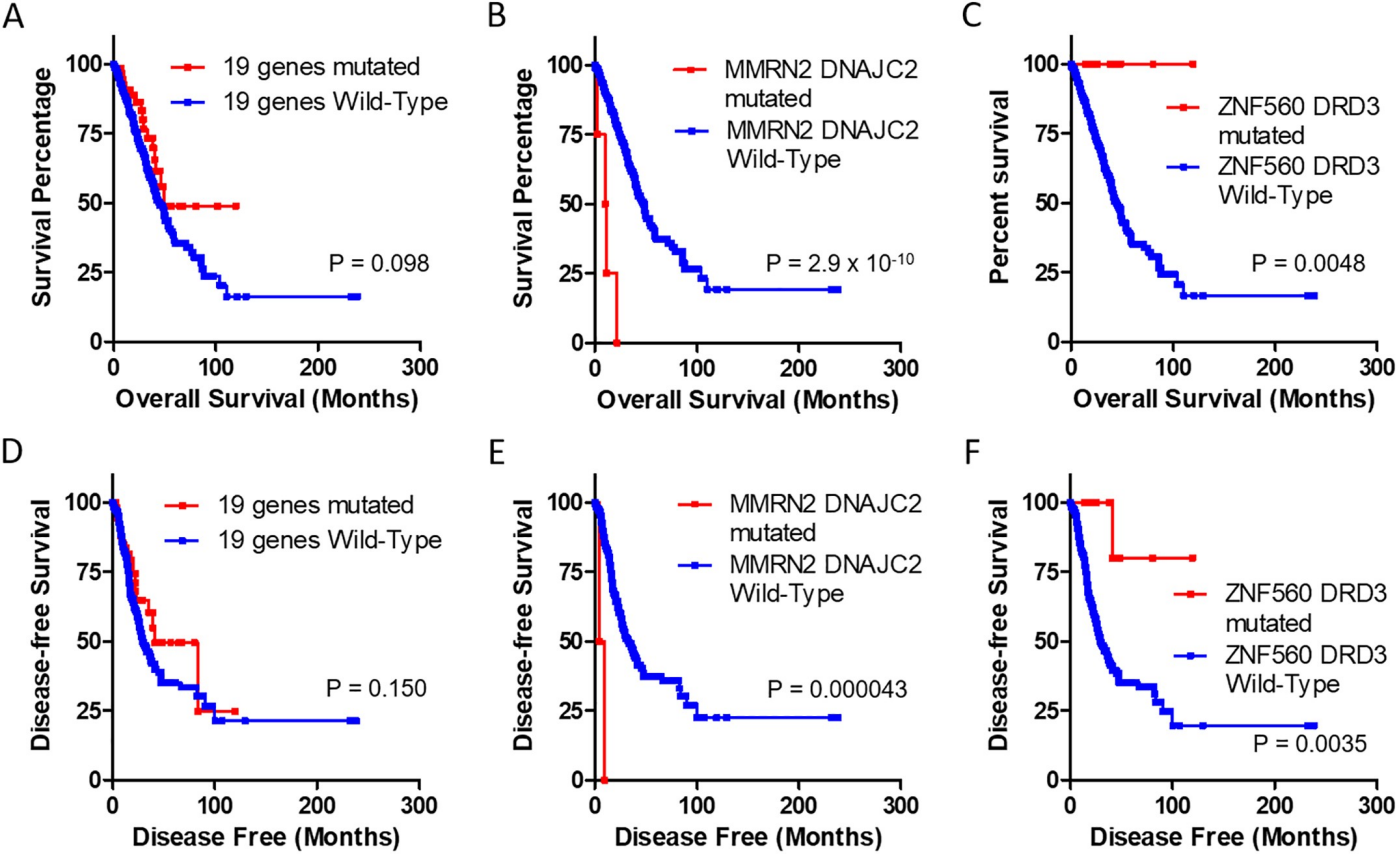
Survival analysis for mutations in 19 genes and survival-related genes. Survival and disease-free survival time of patients with or without specific gene mutations were analyzed using Kaplan-Meier curves. These 19 gene mutations, two gene mutations negatively associated with survival (*DNAJC2* or *MMRN2*), and two gene mutations positively associated with survival (*DRD3* or *ZNF560*) were analyzed. Overall survivals is shown in A-C while disease-free survival is shown in D-F. Red lines indicate mutation positive while blue lines indicate mutation negative. P-values were obtained from Log rank test.

**Fig 3:**
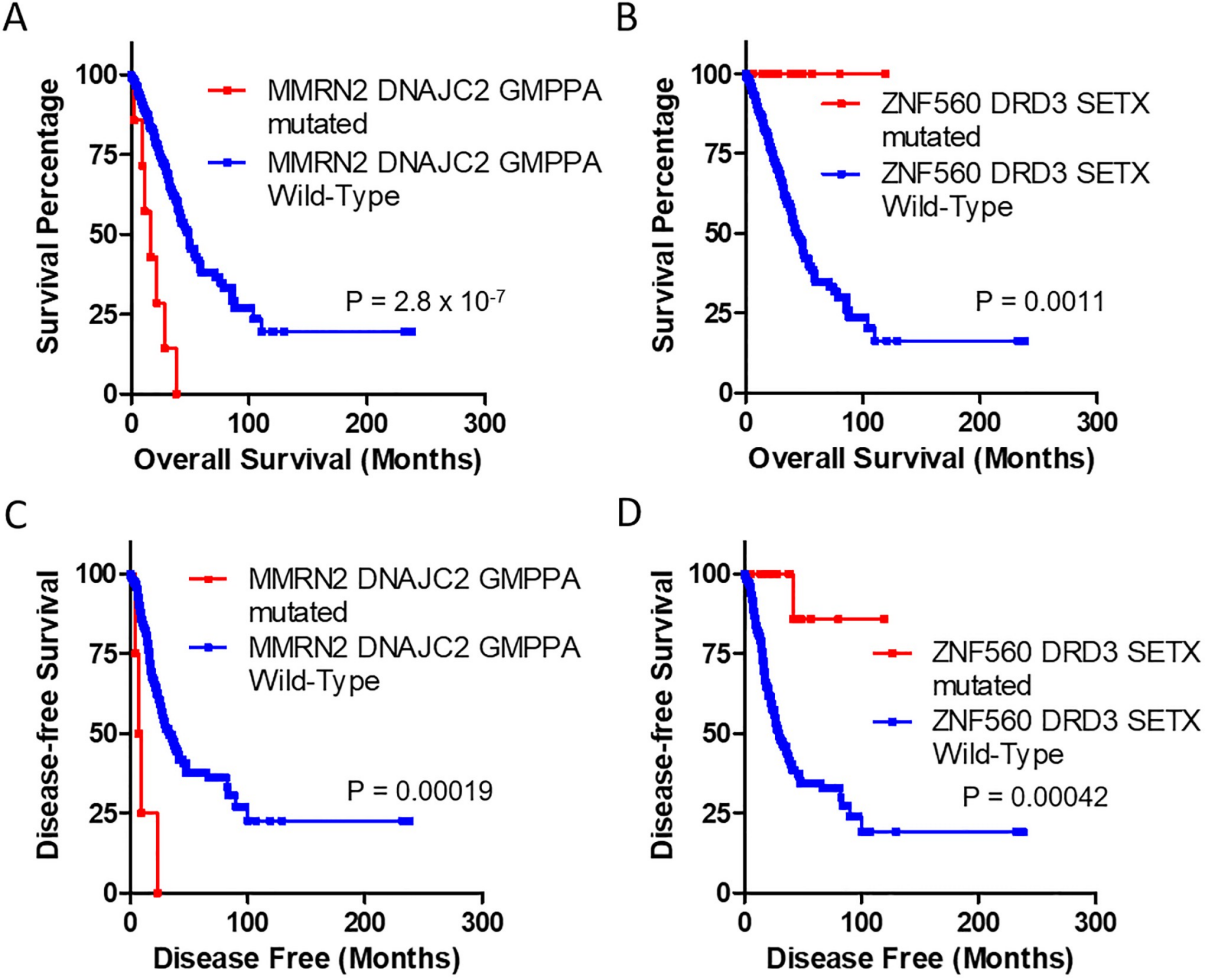
Comparative survival analysis for mutations in survival-related genes. Survival and disease-free survival time of patients with or without specific gene mutations were analyzed using Kaplan-Meier curves. Mutations in three genes (*DNAJC2*, *GMPPA*, or *MMRN2*) negatively associated with survival and mutations in three genes (*DRD3*, *SETX*, or *ZNF560*) positively associated with survival were analyzed. Overall survival is shown in A and B while disease-free survival is shown in C and D. Red lines indicate mutation positive while blue lines indicate mutation negative. P-values were obtained from Log rank test.

To evaluate the association of mutations in multiple genes and survival, mutations in 19 genes and those in genes associated with survival were analyzed (Fig 2 and S2 Table). Mutations in these 19 genes were not associated with overall survival or disease-free survival. Mutations in *DNAJC2* or *MMRN2* were negatively associated with survival. They significantly decreased the median survival time to 9.95 months and the median disease-free survival time to 4.57 months. However, mutations in *DRD3* or *ZNF560* were positively associated with survival. They significantly increased both survival time and disease-free survival time. Furthermore, mutations in *DNAJC2*, *GMPPA*, or *MMRN2* significantly decreased median survival time to 11.27 months and the median disease-free survival time to 6.87 months (Fig 3). Mutations in *DRD3*, *SETX*, or *ZNF560* significantly increased both survival time and disease-free survival time. Patients with LUAD who had mutations in *DNAJC2*, *GMPPA*, or *MMRN2* exhibited significantly shorter survival and earlier recurrence than those without mutations. However, patients with mutations in *DRD3*, *SETX*, or *ZNF560* exhibited longer survival and later recurrence than those without these mutations.

The frequency of mutations in six gene associated with survival was further analyzed in other lung cancer types such as lung squamous cell carcinoma, small cell lung cancer, and another two data sets of LUAD (Table 3 and S3 Fig). Mutations in these six genes were found not only in another two LUAD data sets (n = 21 and n = 4), but also in lung squamous cell carcinoma (n = 28), small cell lung cancer (n = 12), and Pan-lung cancer (n = 146).

**Table 3:** Comparative analysis of mutation frequencies of lung adenocarcinoma-associated six genes in six data sets of different lung cancer cohorts.

Data set \ Gene name	GMPPA	DRD3	DNAJC2	SETX	MMRN2	ZNF560
Lung Adenocarcinoma TCGA (n = 471)	3 (0.64%)	5 (1.06%)	2 (0.42%)	9 (1.91%)	3 (0.64%)	9 (1.91%)
Pan-Lung Cancer Nat Genet 2016 (n = 954)	10 (1.05%)	33 (3.46%)	12 (1.26%)	44 (4.61%)	10 (1.05%)	37 (3.88%)
Lung Squamous Cell Carcinoma TCGA (n = 498)	2 (0.40%)	5 (1.00%)	0 (0.00%)	10 (2.01%)	0 (0.00%)	11 (2.21%)
Small Cell Lung Cancer U Cologne, Nature 2015 (n = 101)	2 (1.98%)	3 (2.97%)	1 (0.99%)	5 (4.95%)	1 (0.99%)	0 (0.00%)
Lung Adenocarcinoma Broad, Cell 2012 (n = 135)	1 (0.74%)	7 (5.19%)	0 (0.00%)	4 (2.96%)	4 (2.96%)	5 (3.70%)
Lung Adenocarcinoma MSKCC 2015 (n = 34)	0 (0.00%)	0 (0.00%)	0 (0.00%)	1 (2.94%)	0 (0.00%)	3 (8.82%)
Sum	18 (0.82%)	53 (2.42%)	15 (0.68%)	73 (3.33%)	18 (0.82%)	65 (2.96%)

Associations of mutations in six genes with survival were analyzed using three data sets of lung cancer cohorts with survival information (Table 4). Mutations in *GMPPA*, *DNAJC2*, and *MMRN2* were significantly associated with patient mortality in lung adenocarcinoma of Pan-Lung Cancer cohort. Mutations in *DNAJC2* and *MMRN2* were significantly associated with shortened overall survival (median survival of 10 and 21.9 months, respectively) (S4 Fig). Mutations in *DRD3* and *SETX* were significantly associated with patient survival, and mutations in *SETX* extended overall survival. These gene mutations were not associated with mortality or overall survival in other types of lung cancer. These results suggested that mutations in *DNAJC2*, *GMPPA*, *MMRN2*, *DRD3*, and *SETX* could be significantly associated with survival in patients with LUAD. They might be considered as biomarkers for predicting survival or recurrence in patients with LUAD.

**Table 4:** Comparative analysis of six-gene mutation frequency and survival in three data sets of lung cancer cohorts.

Chromosome			2	3	7	9	10	19
Mutated gene			GMPPA	DRD3	DNAJC2	SETX	MMRN2	ZNF560
Pan-Lung Cancer Nat Genet 2016 (n = 954)	Mutation	Total	10 (1.05%)	33 (3.46%)	12 (1.26%)	44 (4.61%)	10 (1.05%)	37 (3.88%)
		Living	5	25	6	37	3	28
		Deceased	5	8	6	7	7	9
		Fisher’s exact	0.158	0.697	0.111	0.061	0.0075*	0.711
	Overall Survival	P-value	0.12	0.757	0.0066**	0.063	0.00014***	0.89
		Median Months	28.1	31.51	14.1	63.59	15	39.1
Pan-Lung Cancer Nat Genet 2016; Lung Adenocarcinoma (n = 481)	Mutation	Total	6 (1.25%)	14 (2.91%)	5 (1.04%)	22 (4.57%)	8 (1.66%)	18 (3.74%)
		Living	2	14	1	21	3	16
		Deceased	4	0	4	1	5	2
		Fisher’s exact	0.034*	0.027*	0.014*	0.023*	0.024*	0.27
	Overall Survival	P-value	0.074	0.16	0.00014***	0.027*	0.0025**	0.65
		Median Months	28.1	NA	14.1	NA	21.9	NA
Lung Squamous Cell Carcinoma TCGA (n = 498)	Mutation	Total	2 (0.40%)	5 (1.00%)	0	10 (2.01%)	0	11 (2.21%)
		Living	1	2	NA	6	NA	5
		Deceased	1	3	NA	4	NA	6
		Fisher’s exact	1	0.657	NA	1	NA	0.545
	Overall Survival	P-value	0.33	0.107	NA	0.919	NA	0.747
		Median Months	1.71	9.82	NA	63.5	NA	30.09
Small Cell Lung Cancer U Cologne, Nature 2015 (n = 101)	Mutation	Total	2 (1.96%)	3 (2.94%)	1 (0.98%)	5 (4.90%)	1 (0.98%)	0
		Living	1	1	1	3	1	NA
		Deceased	1	2	0	2	0	NA
		Fisher’s exact	1	1	0.333	0.33	0.333	NA
	Overall Survival	P-value	0.722	0.812	0.266	0.159	0.351	NA
		Median Months	3	27	NA	72	NA	NA

Fisher’s exact P-value: Log rank test, NA: not available,

* indicates p<0.05,

** indicates p<0.01,

*** indicates p<0.001

## Discussion

Lung cancer is the second most common cancer. It has a high mortality rate. The discovery of biomarkers that can predict overall survival of lung cancer patients is essential for treatment of patients. Identification of survival-specific gene mutations is important not only for understanding genetic disparities associated with survival, but also for predicting the survival of LUAD patients. These gene mutations can be significant biomarkers for LUAD. In this study, TCGA LUAD data set was used to derive gene mutations by machine learning. Patients with LUAD were divided into surviving and non-surviving groups and machine learning was performed with four feature selection methods to identify gene mutations associated with survival of patients with LUAD from mutations in about 40,000 genes [16,19–24]. The most frequently observed mutations determined by machine learning were in *SETX* and *ZNF560* genes. Mutational incidence of *SETX*, *ZNF560*, *GMPPA*, and *MMRN2* was significant. Mutations in *MMRN2* and *DNAJC2* were significantly and negatively associated with patient survival while those in *ZNF560* and *DRD3* were positively associated with patient survival. Mutations in genes determined by machine learning seem to influence survival in LUAD.

Because of the relatively small number of mutations in LUAD cohort, mutations in six genes were applied to other data sets of lung cancer cohorts to analyze mutation frequencies and association with survivals such as Lung Adenocarcinoma (Broad and MSKCC) cohorts, Lung Squamous Cell Carcinoma TCGA cohort, Pan-Lung Cancer cohort, and Small Cell Lung Cancer cohort. The average frequency of mutations in six genes was 0.81% ~ 3.30% and their associations with survival were similar between LUAD cohort and Pan-Lung Cancer cohort. Data set of Pan-Lung Cancer cohort was composed of LUAD and lung squamous cell carcinoma. In LUAD of Pan-Lung Cancer cohort, mutations in *DNAJC2*, *GMPPA*, *MMRN2*, *DRD3*, and *SETX* were significantly associated with survival status, and those in *DNAJC2*, *MMRN2*, and *SETX* were significantly associated with overall survival. This result supports that mutations in these six genes can predict the survival of patients with LUAD and overall survival time. They could be considered as biomarkers of LUAD.

Mutations in *MMRN2* and *DNAJC2* were observed to be important for predicting the survival and prognosis negatively. *MMRN2* encodes a multimerin2 which is an elastin microfibril interface-located (EMILIN)-like protein, extracellular matrix glycoprotein [25]. MMRN2 acts as a modified growth factor β antagonist. It can interfere with VEGF-A/VEGFR2 pathway in endothelial cells [25]. Recent studies have demonstrated that CLEC14A-MMRN2 binding has potential for future anti-angiogenic therapy because it plays a role in inhibiting angiogenesis during tumor growth [26]. The *DNAJC2* gene encodes a phosphorylated protein with a J-domain and a Myb DNA-binding domain. Its protein is observed in both nucleus and cytoplasm [27]. DNAJC2 protein can form a heterodimeric complex with the ribosome to acts as a molecular protector for the initial polypeptide chain when exiting the ribosome [27]. DNAJC2 protein has been identified as a leukemia-associated antigen. Its expression is increased in those with leukemic seizures [28]. In addition, chromosomal abnormalities involving the *DNAJC2* gene are associated with primary head and neck squamous cell tumors [29]. These studies have revealed molecular mechanisms that *MMRN2* and *DNAJC2* either cause or exacerbate cancers. However, further studies are needed to determine the role of *MMRN2* and *DNAJC2* in LUAD.

Mutations in *ZNF560* and *SETX* were observed to be important for predicting the survival and prognosis positively. The *ZNF560* gene has been reported in colorectal cancer studies [30]. Left-sided colon cancer (LSCC) and right-sided colon cancer (RSCC) differ in their genetic susceptibilities to neoplastic transformation. Patients with LSCC had low mortality and improved overall 5-year survival rate than patients with RSCC [30]. *ZNF560* was down-regulated in LSCC compared to that in RSCC. It may be useful for predicting a positive prognosis. *SETX* is a RNA/DNA helicase that splices RNA, regulates gene expression, terminates transcription, and stabilizes telomere and genome [31]. Mutations in SETX are linked to neurodegenerative disorders, ataxia oculomotor apraxia type 2, and amyotrophic lateral sclerosis type 4 [31]. Although the role of *SETX* in cancer has not been known, its expression level is relatively lower than other genes. Since there is little research on the role of *ZNF560* and *SETX* in cancers, more researches are needed to understand their roles in LUAD.

Since this study focused on the yield and selection of gene mutation rather than deducing an efficient algorithm through machine learning, a dependent algorithm was used. In this case, the weighted results of the independent algorithm could not be obtained. Utilizing 100% trained data is closer to probability statistics than machine learning. Of 19 gene mutations, six gene mutations were significantly associated with survival in LUAD, showing a relatively high rate (about 32%). Further study is needed to determine the differences between using dependent and independent algorithms in machine learning methods for analyzing medical information of solid tumors.

It is important to apply the optimal feature selection method that classifies human cancer genetic mutations according to specific factors among various feature selection methods. Previously reported feature selection methods in medical studies have used Weka that can implement information gain, correlation, and ranker algorithms, and ensemble learning methods [32–34]. However, in order to classify gene mutations using dependent algorithm, selection methods for prediction of economic demand as well as the above feature selection method were applied to feature selection in this study. Of combination algorithms used, the combination with the highest classification prediction rate was information gain-Naïve bayes combination. It can be adopted to analyze RNA sequence or other medical information in LUAD.

In summary, machine learning was conducted to obtain information necessary to select mutations in genes associated with survival of patients with LUAD. We identified specific mutational markers associated with survival of patients with LUAD. Mutations in *DNAJC2*, *GMPPA*, and *MMRN2* can be used as biomarkers of negative prognosis for patient’s overall survival and disease-free survival while mutations in *DRD3*, *SETX*, and *ZNF560* can be used as biomarkers of positive prognosis. This study also suggested a predictive classification model of LUAD based on mutation expression.

## Supporting information

S1 Fig Machine learning method for gene extraction. The general machine learning method is Method I and our machine learning method is Method II. FS indicates feature selection.
(JPG)

S2 Fig Survival analysis for mutations in survival-related six genes. Survival and disease-free survival time of patients with or without specific gene mutations were analyzed using Kaplan-Meier curves. Overall survival is shown in the left column while disease-free survival is shown in the right column. P-values were obtained from Log rank test.
(JPG)

S3 Fig Comparison of mutation frequencies of lung adenocarcinoma-associated six genes in six lung cancer data sets. Mutations in six genes were demonstrated as frequency (percent).
(JPG)

S4 Fig Survival analysis for mutations in survival-related six genes in adenocarcinoma of Pan-Lung Cancer. Overall survival time of patients with or without specific gene mutations were analyzed using Kaplan-Meier curves. P-values were obtained from Log rank test.
(JPG)

S1 Table Accuracy, precision, recall, and classification error of feature selection. (DOCX)

S2 Table Comparative analysis of overall survival and disease-free survival in the mutation group (two or three genes) and non-mutation group. (DOCX)
