# Supplementary figures and images for: Association of specific gene mutations derived from machine learning with survival in lung adenocarcinoma
Source: PLoS One. 2018 Nov 12;13(11):e0207204. doi: 10.1371/journal.pone.0207204 (PMC6231670; doi:10.1371/journal.pone.0207204)

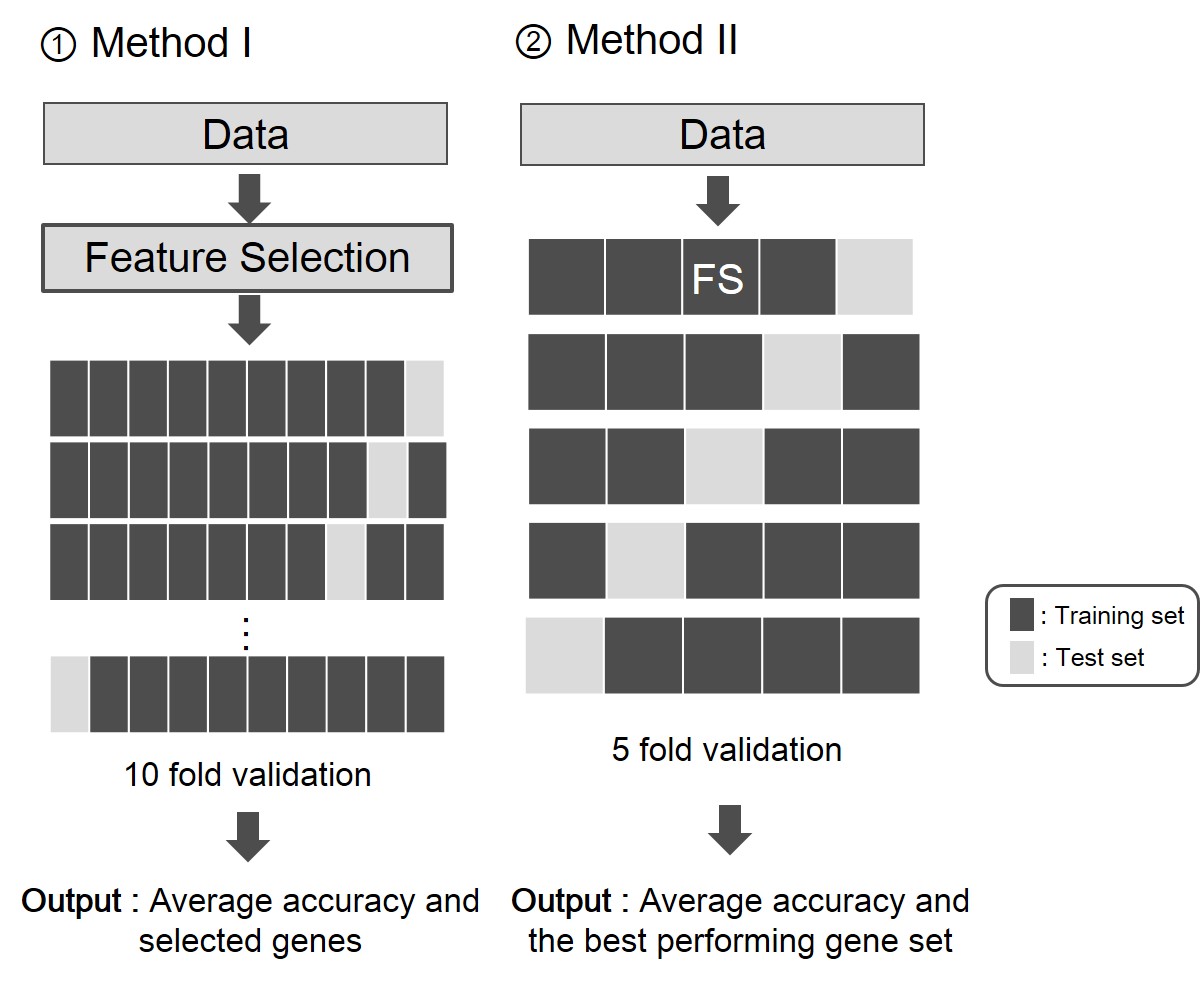

Supplement: S1 Fig — The general machine learning method is Method I and our machine learning method is Method II. FS indicates feature selection. (JPG) [file pone.0207204.s001.jpg]

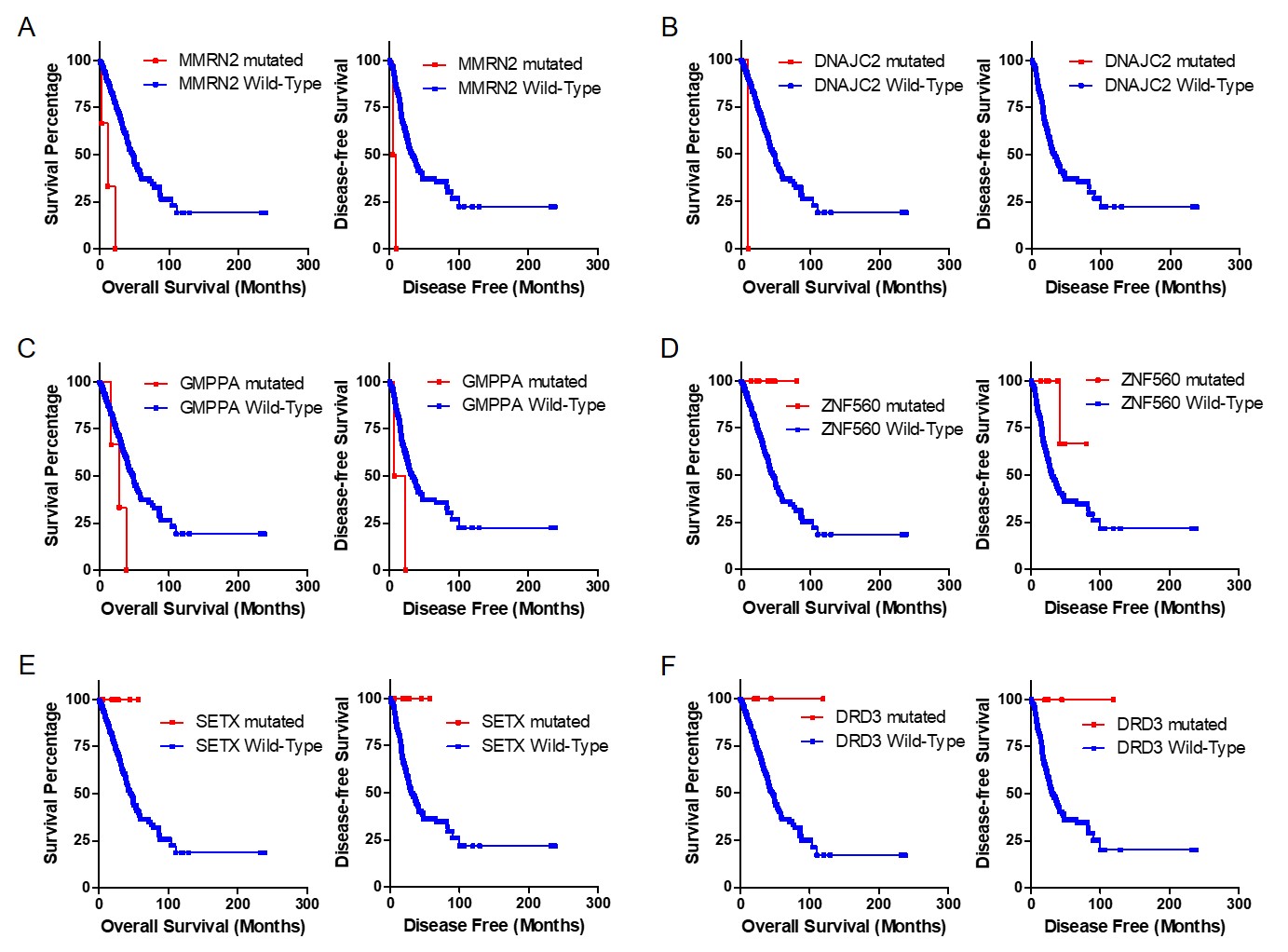

Supplement: S2 Fig — Survival and disease-free survival time of patients with or without specific gene mutations were analyzed using Kaplan-Meier curves. Overall survival is shown in the left column while disease-free survival is shown in the right column. P-values were obtained from Log rank test. (JPG) [file pone.0207204.s002.jpg]

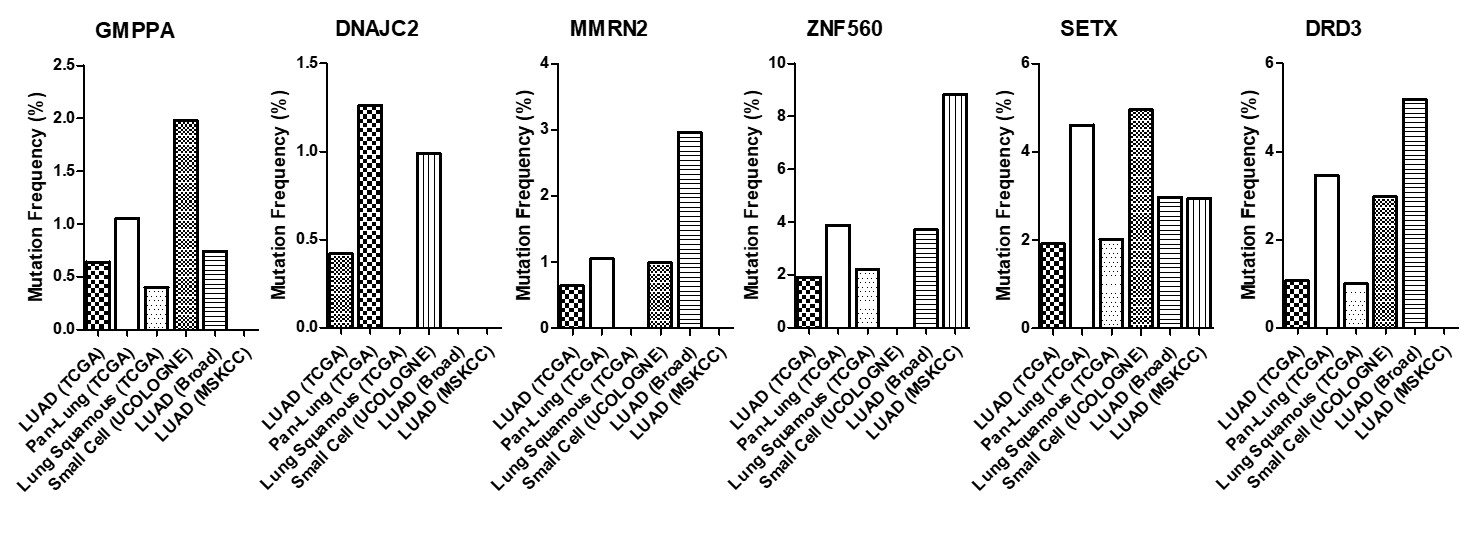

Supplement: S3 Fig — Mutations in six genes were demonstrated as frequency (percent). (JPG) [file pone.0207204.s003.jpg]

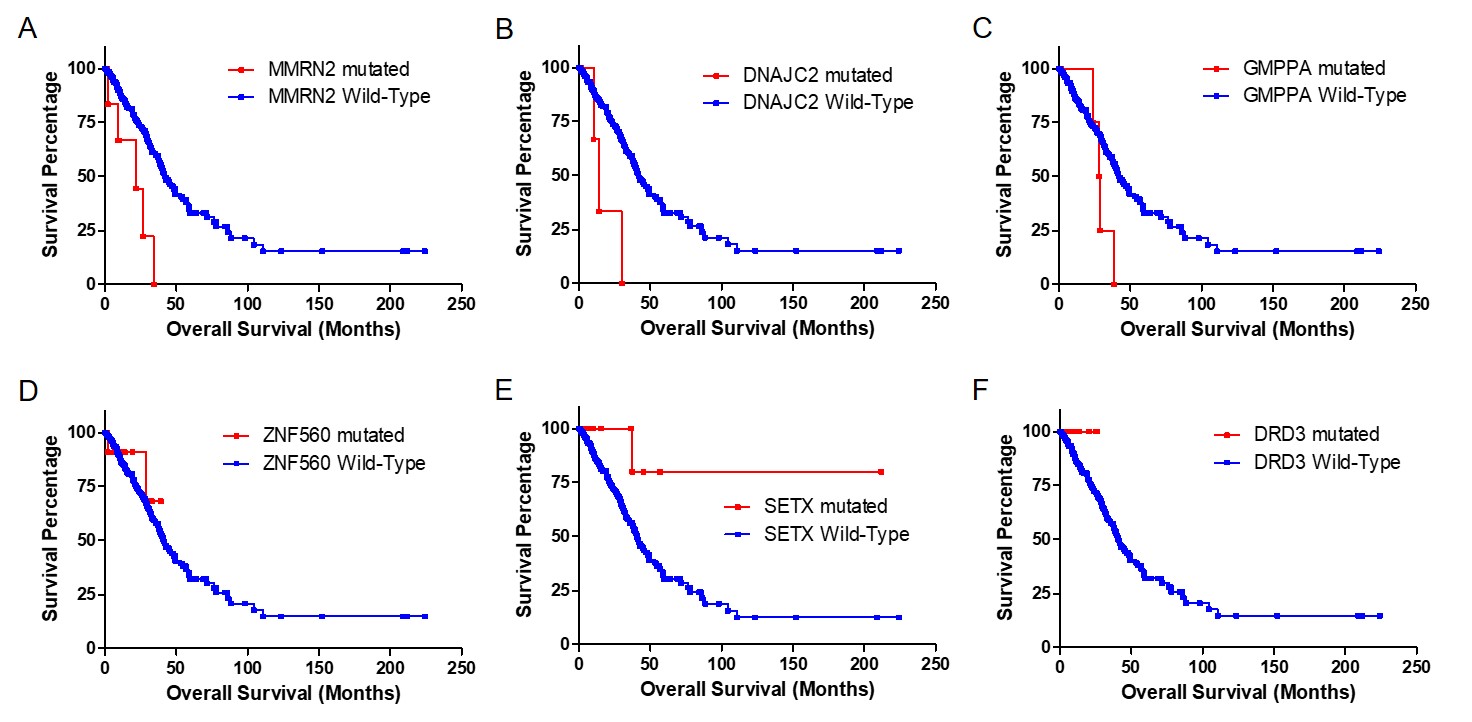

Supplement: S4 Fig — Overall survival time of patients with or without specific gene mutations were analyzed using Kaplan-Meier curves. P-values were obtained from Log rank test. (JPG) [file pone.0207204.s004.jpg]
