# Supplementary material for: Association of specific gene mutations derived from machine learning with survival in lung adenocarcinoma
Source: PLoS One. 2018 Nov 12;13(11):e0207204. doi: 10.1371/journal.pone.0207204 (PMC6231670; doi:10.1371/journal.pone.0207204)
Supplement: S1 Table — (DOCX) [file pone.0207204.s005.docx]

S1 Table. Accuracy, precision, recall, and classification error of feature selection

| **Feature**  **selection** | **Classification** | **Optimize** | | | |
| --- | --- | --- | --- | --- | --- |
|  |  | **Accuracy** | **Precision** | **Recall** | **Classification Error** |
| **Information Gain** | **Naïve Bayes** | 88.11% | 86.76% | 87.89% | 11.89% |
|  | **K-NN** | 70.81% | 79.56% | 59.60% | 29.19% |
|  | **SVM** | 69.46% | 70.60% | 59.22% | 30.54% |
|  | **Decision Tree** | 68.92% | 72.78% | 57.62% | 31.08% |
| **Chi-squared** | **Naïve Bayes** | 85.95% | 89.22% | 80.98% | 14.05% |
|  | **K-NN** | 71.08% | 75.20% | 60.82% | 28.92% |
|  | **SVM** | 71.89% | 74.00% | 62.63% | 28.11% |
|  | **Decision Tree** | 68.65% | 71.52% | 57.41% | 31.35% |
| **MRMR** | **Naïve Bayes** | 76.49% | 80.15% | 68.56% | 23.51% |
|  | **K-NN** | 68.11% | 83.43% | 55.30% | 31.89% |
|  | **SVM** | 71.35% | 81.37% | 60.19% | 28.65% |
|  | **Decision Tree** | 68.11% | 77.97% | 55.64% | 31.89% |
| **Correlation** | **Naïve Bayes** | 85.68% | 89.04% | 80.60% | 14.32% |
|  | **K-NN** | 71.08% | 75.20% | 60.82% | 28.92% |
|  | **SVM** | 72.43% | 70.95% | 65.75% | 27.57% |
|  | **Decision Tree** | 68.65% | 71.52% | 57.41% | 31.35% |
