# Supplementary material for: Association of specific gene mutations derived from machine learning with survival in lung adenocarcinoma
Source: PLoS One. 2018 Nov 12;13(11):e0207204. doi: 10.1371/journal.pone.0207204 (PMC6231670; doi:10.1371/journal.pone.0207204)
Supplement: S2 Table — (DOCX) [file pone.0207204.s006.docx]

S2 Table. Comparative Analysis of Overall Survival and Disease-Free Survival in the Mutation Group (two or three genes) and Non-mutation Group

| **Mutated gene** | | **19 genes** | **DNAJC2, MMRN2** | **DNAJC2, MMRN2, GMPPA** | **ZNF560, DRD3** | **ZNF560, DRD3, SETX** |
| --- | --- | --- | --- | --- | --- | --- |
| Mutation | Total | 62 | 5 | 8 | 14 | 23 |
|  | Living | 45 | 0 | 0 | 14 | 23 |
|  | Deceased | 17 | 5 | 8 | 0 | 0 |
|  | Fisher's  exact | 0.315 | 0.0056 | 0.0002 | 0.003 | 0.0001 |
| Overall Survival | P-value | 0.098 | 0.00000000029 | 0.00000028 | 0.0048 | 0.0011 |
|  | Median Months | 49.01 | 9.95 | 11.27 | NA | NA |
| Disease Free Survival | P-value | 0.150 | 0.000043 | 0.00019 | 0.0035 | 0.00042 |
|  | Median Months | 41.33 | 4.57 | 6.87 | NA | NA |
